# A brief comment on the predictive value of myeloperoxidase-conjugated DNA level in patients with septic shock

**DOI:** 10.1186/s13054-018-2246-z

**Published:** 2018-11-13

**Authors:** Xu Li, Xiaochun Ma

**Affiliations:** Department of Critical Care Medicine, The First Affiliated Hospital, China Medical University, North Nanjing Street 155, Shenyang, 110001 Liaoning Province China

We read with great interest the recent article by Maruchi et al., “Plasma myeloperoxidase-conjugated DNA level predicts outcomes and organ dysfunction in patients with septic shock” [1], in which the authors conducted a concise study of the predictive value of plasma myeloperoxidase (MPO)-conjugated DNA level in septic shock patients. MPO-DNA and cell-free DNA (cf-DNA) are components of neutrophil extracellular traps (NETs), which play an important role in the process of sepsis. However, the effects of NETs and the precise mechanism are still a matter of debate [2]. We would like to offer the following comments.

First, the authors did not list the preexisting diseases and treatments for the septic shock patients. Circulating NETs can also increase in other diseases as described in the discussion. Septic shock patients are often treated with fluid resuscitation, anticoagulants, and so on. Fluid resuscitation may dilute the plasma levels of MPO-DNA and cf-DNA. Anticoagulation may also affect the results. So it’s important to exclude the influence of these factors on the results. Second, the authors studied the correlation of MPO-DNA and cf-DNA levels on days 3 and 7 with organ failure parameters but reported the correlation of MPO-DNA and cf-DNA levels on days 1 and 7 with coagulation parameters. What is the basis for choosing the time points? Why not study the correlations on all three days? NET formation in the early stage of septic shock is involved in pathogen recognition and preventing bacterial dissemination in sepsis, which is beneficial to the host [3]; however, uncontrolled activation during septic shock can be deleterious. Though day 1 may not be the early stage for patients, it is the earliest time for clinicians to get samples. Hence, we are interested in the correlations of MPO-DNA and cf-DNA levels on day 1 with organ failure and coagulation parameters. Third, the authors showed that neither MPO-DNA nor cf-DNA had correlations with coagulation parameters. In the trial by Delabranche et al. [4] plasma MPO-DNA levels in septic disseminated intravascular coagulation (DIC) patients were increased compared with patients without DIC. It is apparent that data in the current literature on the predictive value of circulating NETs in septic patients are variable and often conflicting. Theoretically, NETs take part in the host’s immune defense and coagulation activation in sepsis [5] and plasma NET levels should be elevated in septic DIC patients. Although the number of DIC patients is limited, results from this subgroup would be interesting.

We think, therefore, that further clinical trials regarding the predictive effect of circulating NETs in septic patients are needed.

## Abbreviations

cf-DNA: Cell-free DNA
DIC: Disseminated intravascular coagulation
IL: Interleukin
MPO: Myeloperoxidase
NET: Neutrophil extracellular trap
